# 4-Hydroxynonenal regulates mitochondrial function in human small airway epithelial cells

**DOI:** 10.18632/oncotarget.6131

**Published:** 2015-10-15

**Authors:** Lakshmi Galam, Athena Failla, Ramani Soundararajan, Richard F. Lockey, Narasaiah Kolliputi

**Affiliations:** ^1^ Division of Allergy and Immunology, Department of Internal Medicine, Morsani College of Medicine, University of South Florida, Tampa, FL, USA

**Keywords:** acute lung injury, hyperoxia, ROS, 4-HNE, mitochondrial dysfunction, Immunology and Microbiology Section, Immune response, Immunity

## Abstract

Prolonged exposure to oxidative stress causes Acute Lung Injury (ALI) and significantly impairs pulmonary function. Previously we have demonstrated that mitochondrial dysfunction is a key pathological factor in hyperoxic ALI. While it is known that hyperoxia induces the production of stable, but toxic 4-hydroxynonenal (4-HNE) molecule, it is unknown how the reactive aldehyde disrupts mitochondrial function. Our previous *in vivo* study indicated that exposure to hyperoxia significantly increases 4-HNE-Protein adducts, as well as levels of MDA in total lung homogenates. Based on the *in vivo* studies, we explored the effects of 4-HNE in human small airway epithelial cells (SAECs). Human SAECs treated with 25 μM of 4-HNE showed a significant decrease in cellular viability and increased caspase-3 activity. Moreover, 4-HNE treated SAECs showed impaired mitochondrial function and energy production indicated by reduced ATP levels, mitochondrial membrane potential, and aconitase activity. This was followed by a significant decrease in mitochondrial oxygen consumption and depletion of the reserve capacity. The direct effect of 4-HNE on the mitochondrial respiratory chain was confirmed using Rotenone. Furthermore, SAECs treated with 25 μM 4-HNE showed a time-dependent depletion of total Thioredoxin (Trx) proteins and Trx activity. Taken together, our results indicate that 4-HNE induces cellular and mitochondrial dysfunction in human SAECs, leading to an impaired endogenous antioxidant response.

## INTRODUCTION

Hyperoxic acute lung injury (HALI) triggers cellular damage and produces reactive oxygen species (ROS) that interfere with the cell's intrinsic antioxidant responses [1-3]. During oxidative stress, the cell will undergo lipid peroxidation reactions, in which destructive oxidized by-products are produced, such as 4-hydroxynonenal (4-HNE) and malondialdehyde (MDA) [4]. 4-HNE is an endogenous α,β-unsaturated hydroxyalkenal that is produced in a concentration range of 0.1-3 μM under physiological conditions [5]. However, under oxidative stressful conditions, the concentration of 4-HNE that accumulates in membranes ranges from 10 μM to 5 mM [5]. In response to hyperoxia-induced oxidative stress, physiological 4-HNE levels are measured to be less than 20 ng/mg protein [2]. 4-HNE can additionally induce protein dysfunction by forming adducts with lysine, histidine, and cysteine residues, as well as generating stable Michael addition products and disulfide bonds [5, 6]. These detrimental reactions further lead to 4-HNE-induced cellular apoptosis and death [7, 8].

Previously, we have demonstrated that hyperoxia induces ROS production in mice and affects lung mitochondrial morphology [9]. ROS production is well known to enhance lipid peroxidation reactions, damaging proteins within the mitochondrial respiratory chain and reducing oxygen consumption rate [8, 10-12]. Interestingly, it has been shown that Thioredoxin (Trx) protein systems mediate protection against oxidative stress induced by hyperoxic lung injury in mice and are critical for cellular survival [13, 14]. Trx proteins endogenously protect and reinstate oxidized cellular proteins, and can perform enzymatic reduction processes, acting as a proteasome response that reduces disulfide protein bonds formed by thiol redox reactions [14-17]. Trx system proteins ensure proper protein folding, mediate transcription factor redox reactions, and maintain mitochondrial membrane potential, all important defensive responses to oxidative stress [13, 17]. However, Trx serves as a prime target for 4-HNE because these proteins contain thiol-disulfide bonds composed of cysteine residues [10, 13, 16, 18].

Thus far, the effects of 4-HNE have not been explored in human SAECs. SAECs have an important role in the lungs as they serve as a barrier to external pathogens and mediate normal immune function [19]. SAEC cells are also vulnerable to oxidative stress, serving as a critical cell type that should be protected [20, 21]. Hyperoxia-induced oxidative stress is an important model that reproduces the pulmonary-specific damaging effects of ALI, as it disrupts epithelial and endothelial barriers, and generates the release of toxic molecules and pro-inflammatory mediators [1]. Prolonged exposure to oxygen fractions (F_O2_) of 0.8 or greater are shown to induce respiratory distress in small animal models as well as pulmonary edema and proliferative fibrosis in lower order primate models [1]. The link between mechanically supplied oxygen and HALI appeared in the 1960s and progressed when studies demonstrated increases in tissue inflammation and ROS release, which is associated with cellular damage and a secondary inflammatory response to ROS from the activation of macrophages, platelets, and neutrophils [1]. Thus, hyperoxia provides a basis on which to investigate the pathogenesis of pulmonary disease and cellular damage.

We first measured the formation of 4-HNE-Protein adducts and MDA levels in total lung homogenates in mice exposed to hyperoxia. This was followed by an *in vitro* study using human SAECs to characterize the effects of 4-HNE and determine its functionality. We demonstrate an elevation of 4-HNE in SAECs and predict that mitochondrial function will be reduced. Furthermore, we hypothesize that Trx proteins will be modified in the presence of 4-HNE, impairing an ROS-mediated protective response present in mitochondria. Our results indicate that 4-HNE decreases human SAEC viability with an increase in cleaved caspase-3 activity. 4-HNE augmented the production of mitochondrial ROS, followed by a reduction in mitochondrial oxygen consumption. Mitochondrial membrane potential and enzymatic processes were directly affected by 4-HNE. 4-HNE was also shown to inhibit mitochondrial antioxidant mechanisms by depleting total Trx and inhibiting Trx activity, further contributing to cellular dysfunction and death. Supplemental oxygen therapy is commonly administered to patients suffering from hypoxia and a range of diseases; thus, they are susceptible to lung oxygen toxicity [22-24]. Therefore, this study aims to provide insight into promising alternative therapeutic applications that reduce 4-HNE effects on human SAECs during hyperoxic lung injury.

## RESULTS

### Hyperoxia induces the formation of 4-HNE-protein adducts

**Figure 1:**
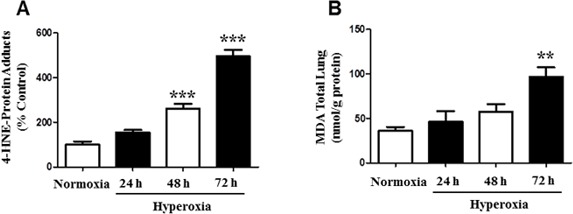
Formation of 4-HNE-Protein Adducts and MDA levels in mice Male and female C57BL/6J mice (*N* = 6) were exposed to normoxia and hyperoxia for 24, 48, and 72 hours. **A.** Mice lung homogenates were isolated and 4-HNE-Protein adduct levels were measured by ELISA with primary anti-HNE His antibody and secondary horseradish peroxidase antibody. Values are expressed as percent of control. **B.** Total lung MDA levels (nmol/g protein) were determined by a MDA Lipid Peroxidation and quantified by reading optical density at 532 nm. Data are shown as means ± SEM. **p-value < 0.01 *vs*. control, ***p-value < 0.001 *vs*. control.

Oxidative stress causes cellular and molecular dysfunction [21, 22, 25]. Exposure to hyperoxia stimulates the production of lipid-peroxidation by-products, such as 4-Hydroxynonenal (4-HNE) and MDA [4, 5]. To investigate whether hyperoxia induces elevated 4-HNE levels, we first measured the production of 4-HNE-Protein adducts in mice. Mice (*N* = 6) were exposed to normoxia and hyperoxia for 24, 48, and 72 hours. Compared to room air (normoxia), mice exposed to prolonged hyperoxia showed a significant increase in the levels of 4-HNE-Protein adducts (Figure 1A). Hyperoxia exposure for 24 h did not show a significant difference in 4-HNE-Protein-Adducts relative to the control. However, exposure to 48 h hyperoxia resulted in a 2-fold increase of 4-HNE-Protein-adducts, whereas at 72 h post-hyperoxia exposure, there was a 4 fold increase in 4-HNE-Protein-adducts (Figure 1A). The dose-dependent response indicates that prolonged hyperoxia exposure and oxidative stress leads to post-translational protein modification, impairing normal cellular processes. Furthermore, we measured MDA (nmol/g protein) levels in mice total lung homogenates under the same exposure conditions as 4-HNE (Figure 1B). The results show that prolonged exposure to hyperoxia for 72 h significantly increased MDA levels 2.7-fold, compared to normoxia. Conversely, hyperoxia exposure for 24 and 48 h did not result in significant MDA levels. These results suggest the production of toxic peroxidized lipids in response to hyperoxia-induced oxidative stress.

### 4-HNE induces cellular dysfunction

**Figure 2:**
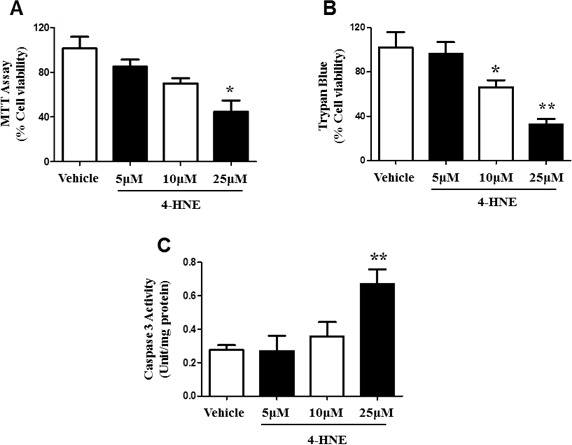
Effects of 4-HNE on SAEC viability and cleaved caspase-3 Cell viability was assessed by **A.** MTT Cell Proliferation and **B.** Trypan Blue Exclusion Assay. SAECs were treated with 5, 10, or 25 μM 4-HNE and incubated for 1 hour, then compared to vehicle controls. For MTT Assay, SAECs were then treated with yellow MTT (0.5 mg/ml) to measure absorbance of purple formazan products at 570 nm. For Trypan Blue Assay, SAECs were then treated with Trypan blue dye (0.04% in PBS), and viable and dead (blue) cells were counted, using a total of 200 cells for standardization. Values are presented as percentage of cell viability. **C.** Caspase-3 activity (Unit/mg protein) was determined by a colorimetric assay in SEACs treated with 5-25 μM 4-HNE, with vehicle cells for comparison. Data is reported as means ± SEM. *p-value < 0.05 *vs*. control, **p-value < 0.01 *vs*. control.

4-HNE is highly cytotoxic and serves as an indicator of oxidative stress [4, 5, 11]. Next, we investigated the functional relevance of 4-HNE in human SAECs. SAECs were investigated since epithelial cells are vulnerable to oxidative damage and permeability of the epithelial membrane barrier can lead to the entry of external pathogens and impairment of the release of regulatory immune factors. Deregulation of SAEC viability was assessed by MTT Cellular Proliferation Assay. SAECs were treated with 5, 10, 25 μM of 4-HNE, and compared to vehicle controls (Figure 2A). There was a dose-dependent decrease in SAEC viability when exposed to 4-HNE which is an indicator of cellular dysfunction. There was significant 2 fold decrease in cellular viability in SAECs exposed to 25 μM 4-HNE, when compared to vehicle-treated controls. We further examined cellular viability by using a Trypan blue exclusion assay. SAECs were treated with the same chemical conditions as in the MTT assay. SAECs treated with 5 μM 4-HNE did not show a significant difference in cell damage (Figure 2B). However, there was a significant decrease in cell viability at 10 and 25 μM 4-HNE. A 3.2-fold decrease in viability was observed with 25 μM 4-HNE. Caspase-3 plays a critical role in the execution of apoptosis and thus serves as a marker for apoptosis activation [26]. Cleaved caspase-3 activity was shown to be increased after 10 μM 4-HNE; however a significant 2.4-fold increase in caspase-3 activity was found with 25 μM 4-HNE treatment (Figure 2C). Taken together, these results indicate that 4-HNE causes cellular dysfunction by decreasing metabolic activity and inducing cell death related mechanisms.

### 4-HNE elicits mitochondrial damage

**Figure 3:**
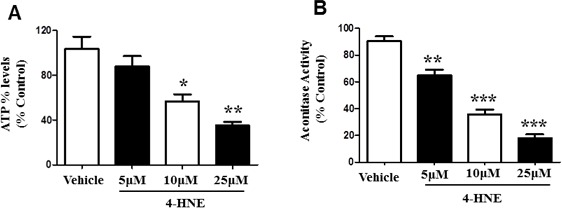
4-HNE reduces SAEC ATP% levels and aconitase activity Cultured SAECs were treated with 5, 10, and 25 μM of 4-HNE and compared to vehicle controls. **A.** Percent ATP levels were determined by lucerifase assay and detection of light production by a luminometer. **B.** Mitochondrial dysfunction was assessed by Aconitase assay. Aconitase activity was determined by measuring NADPH production from conversion of μU of citrate to isocitrate per minute per mg of mitochondrial protein, with absorbance measured at 340 nm. Values are expressed as percent of control. Data is reported as means ± SEM. *p-value < 0.05 *vs*. control, **p-value < 0.01 *vs*. control, ***p-value < 0.0001 *vs*. control.

ATP is a critical form of cellular energy produced during mitochondrial respiration [11]. Oxidative stress increases the concentration of reactive oxygen species and lipid peroxidation by-products, thereby inhibiting mitochondrial respiration [6, 11]. We therefore further investigated SAEC mitochondrial function in the presence of 4-HNE. ATP levels were measured in SAECs treated with 5, 10, 25 μM of 4-HNE, and compared to vehicle-treated control cells (Figure 3A). A concentration dependent response was observed after treatment with 4-HNE and the increase in concentration of 4-HNE lead to a further reduction in ATP levels. A significant 1.8 and 3 fold decrease in ATP levels was observed in response to 10 and 25 μM 4-HNE, respectively. Our results suggest that 4-HNE mediates mitochondrial dysfunction and impairs the synthesis of ATP. We further assessed mitochondrial activity by Aconitase assay (Figure 3B). Aconitase is an enzyme involved in the mitochondrial TCA cycle, a sequence of reactions that is essential for biochemical pathways and ATP synthesis *via* oxidative phosphorylation [27, 28]. SAECs were treated with 5, 10, and 25 μM of 4-HNE and compared to vehicle-treated control cells, and assayed for Aconitase activity. Treatment with 4-HNE produced a significant reduction in Aconitase activity in a concentration dependent response (Figure 3B). Compared to the vehicle-treated control, SAECs treated with 5 μM 4-HNE showed a 30% decrease in Aconitase activity, while treatment with 10 μM or 25 μM lead to a 60% decrease and 80% decrease in Aconitase activity, respectively. These results further confirm 4-HNE-induced mitochondrial dysfunction and support our findings of decreased ATP levels.

### 4-HNE impairs mitochondrial oxygen consumption rate

**Figure 4:**
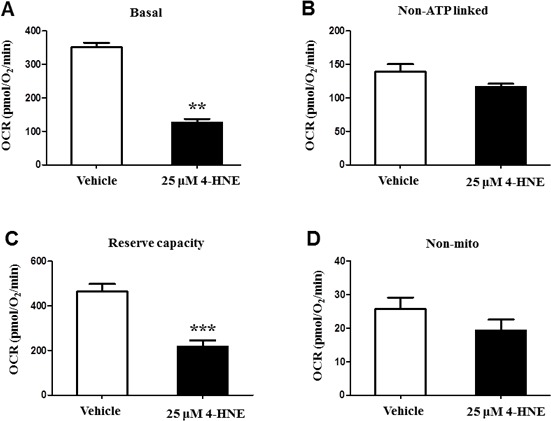
Bioenergetic and oxygen consumption evaluation of SAEC mitochondria SAECs were treated with 25 μM 4-HNE and compared to vehicle controls. **A.** Basal, **B.** non-ATP linked, **C.** reserve capacity, and **D.** non-mitochondrial OCR (pmol/O_2_/min) were measured in SAECs by XF analysis. After basal OCR was measured, oligomycin (1μg/ml), FCCP (1μM), and antimycin A (10 μM) were injected consecutively through Seahorse Flux Pak cartridges, with resulting OCR measured after each injection. Data is reported as means ± SEM. **p-value < 0.01 *vs*. control, ***p-value < 0.001 *vs*. control.

In the presence of 4-HNE, a cell must respond to an increased energy demand, due to a stressful cellular environment [8, 11]. To determine whether 4-HNE impairs mitochondrial oxygen consumption rate OCR, we measured basal, non-ATP linked, maximal, and non-mitochondrial OCR (pmol/O_2_/min) within SAECs (Figure 4A-4D). Each reaction was tested with 25 μM of 4-HNE and compared to a control, since our previous experiments (Figure 2A-2B) showed that 25 μM 4-HNE elicited the greatest response in SAECs. Basal OCR was significantly decreased in response to 4-HNE, as compared to the controls (Figure 4A). After the addition of oligomycin, non-ATP OCR was measured and did not show significant differences between 4-HNE and the control, indicating the dependence of 4-HNE on mitochondrial ATP synthase (Figure 4B). Maximal mitochondrial respiration was induced by addition of FCCP to determine the reserve capacity. Compared to the control, 4-HNE significantly lowered the maximal mitochondrial OCR more than 2-fold, signifying that 4-HNE depletes additional reserved mitochondrial energy (Figure 4C). Non-mitochondrial respiration was assessed with antimycin A (AA) treatment. Control and 4-HNE measurements did not differ with response to OCR (Figure 4D), suggesting 4-HNE effects are not significantly associated with non-mitochondrial OCR. These results indicate that 4-HNE has a direct role in reducing the efficiency of mitochondrial respiration.

### Reduced mitochondrial membrane potential by 4-HNE

**Figure 5:**
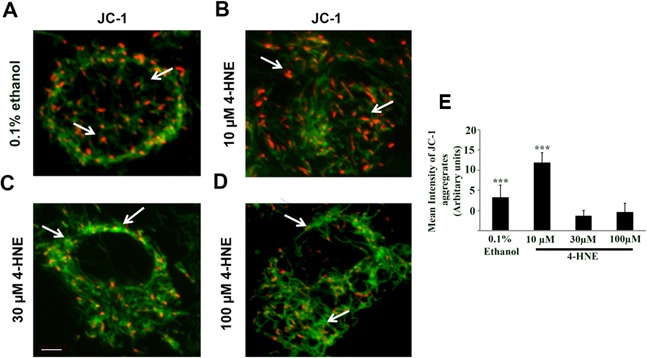
Assessment of mitochondrial dysfunction by JC-1 staining of 4-HNE treated SAECs SAECs were plated at a density of 2 × 10^5^ cells/ml in a 12 mm Nunc glass bottom dish and treated with various concentration of 4-HNE (10-100 μM) or vehicle at 37°C for 15 min. The medium was replaced and cells were incubated with JC-1 probe (10 μg/ml) at 37°C for 15 min, washed and live imaging was performed using confocal microscope. **A.** = Vehicle (0.1% ethanol). **B.**-**D.** = 10, 30 and 100 μM 4-HNE. In vehicle and 10 μM 4-HNE treated sample, predominantly orange-red fluorescence is seen in the mitochondria (arrows in **A.** and **B.**), whereas with 30 and 100 μM 4-HNE treatment, mitochondria showed higher green fluorescent signal relative to vehicle or 10 μM 4-HNE (arrows in **C.** and **D.**). **E.** The JC-1 aggregates were quantified using Image J software and the mean intensity is expressed as Arbitrary Units. ***p < 0.001 Vehicle (0.1% Ethanol) *vs*. 4-HNE (30 and 100 μM), ***p < 0.001 10 μM 4-HNE and 30 and 100 μM 4-HNE, respectively. Scale bar = 10 μm, Magnification = 60X.

To assess whether 4-HNE affected mitochondrial membrane protein function, JC-1 mitochondrial staining was utilized. We then analyzed mitochondrial membrane potential as an indicator of apoptosis and damage by live cell imaging analysis (Figure 5A-5D). A reduction in membrane potential results in diffusion of the dye from the mitochondria and a red to green fluorescence change [9]. SAECs were treated with 10, 30, and 100 μM 4-HNE and compared to vehicle (0.1% ethanol) control cells. Vehicle-treated cells and cells treated with 10 μM 4-HNE show red JC-1 aggregates, represented by arrows in Figure 5A-5B. Treatment with 30 and 100 μM 4-HNE resulted in formation of green JC-1 monomeric aggregates indicating a progressive reduction in membrane potential or depolarization, with arrows in Figure 5B-5D indicating higher green fluorescence. These results indicate that 4-HNE disrupts mitochondrial membrane potential, causing mitochondrial dysfunction. Quantitation of the JC-1 aggregates showed a significant decrease proportional to an increase in 4-HNE treatment (30-100 μM 4-HNE) relative to 10 μM or vehicle (0.1% ethanol) treated control (Figure 5E). These results further support the reduction in ATP levels and impairment of mitochondrial oxygen consumption rate in 4-HNE-treated SAECs.

### Generation of mitochondrial ROS is produced in response to 4-HNE

**Figure 6:**
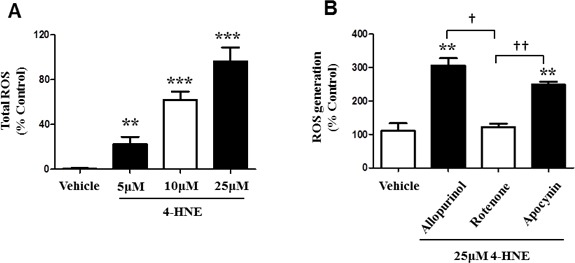
4-HNE induces generation of ROS within SAECs and is specific to the SAEC mitochondrial respiratory chain **A.** SAECs were treated with 5, 10, and 25 μM 4-HNE and compared to vehicle controls. Cells were cultured and treated with 10 μM DCFH-DA for 30 min. The oxidation of DCFH-DA to DCF by ROS was determined by measuring fluorescence intensity at 480 nm (excitation) and 530 nm (emission). **B.** SAECs were then directly treated with 25 μM 4-HNE and compared to vehicle controls. Mitochondrial production of ROS was measured by addition of the inhibitors allopurinol (100μM), rotenone (1 μM), and apocynin (1 mM). Site of ROS production (xanthine oxidase, mitochondrial chain complex I, and NADPH-oxidase) was evaluated. Values are expressed as percent control. Data is reported as means ± SEM. **p-value < 0.01 *vs*. control, ***p-value < 0.001 *vs*. control, † *p*-value < 0.01, ††*p*-value < 0.001.

During oxidative stress, reactive oxygen species accumulate due to the inability of the cell to detoxify them, leading to the production of oxygenated species that can react with protein, inducing cellular damage and effecting energy production [10, 18]. Since our results confirm that 4-HNE inhibits mitochondrial oxygen consumption, we investigated the production of total ROS within SAECs in the presence of 4-HNE. SAECs were treated with 5, 10, 25 μM of 4-HNE and compared to vehicle-treated cells (Figure 6A). All tested concentrations of 4-HNE produced significant increases in ROS activity, when compared to vehicle-treated controls. Vehicle cells produced a negligible amount of ROS, whereas exposure to 5 μM 4-HNE resulted in a 19% increase in ROS activity, at 10μM there was a 59% increase in ROS and at 25μM, it reached a 92% increase in ROS. These results validate that 4-HNE contributes to the production of ROS and cellular damage. We further investigated the origin of 4-HNE generated ROS (Figure 6B). Previously, we found that SAECs treated with 25 μM showed the greatest increase in total ROS. We used this concentration for all subsequent experiments. SAECs were also treated with a specific inhibitor of mitochondrial electron transport chain complex 1 ROS production (Rotenone), as well as inhibitors for alternative cytoplasmic sources of ROS production, namely oxidative xanthine oxidase (Allopurinol) and NADPH-oxidase (Apocynin). Addition of Allopurinol and Apocynin significantly increased ROS production, as compared to vehicle-treated cells. Addition of Rotenone yielded ROS production similar to vehicle-treated control and a significant difference was observed in comparison to Allopurinol and Apocynin treatment. Therefore, only Rotenone reduced 4-HNE-mediated ROS production, suggesting that the process is specific to mitochondrial respiration, which is further supported by the reduction in mitochondrial membrane potential previously observed (Figure 5).

### Time dependent Trx depletion and activity suppression by 4-HNE

**Figure 7:**
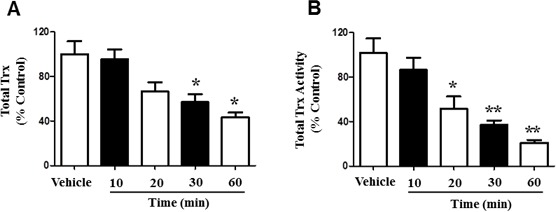
4-HNE affects the function of Trx within SAEC mitochondria **A.** Total Trx depletion and **B.** activity suppression were measured within SAECs. Cells were treated with 25 μM 4-HNE and effects were measured at 10, 20, 30, and 60 min time intervals, and compared to vehicle controls. Cells were lysed with 10.1% Triton X-100/PBS (100 μl). Total Trx activity was then determined by insulin-reducing assay and NADPH oxidation was measured by spectrophotometric analysis at 340 nm over 5 minutes. Values are presented as percentage of controls. Data are expressed as means ± SEM. *p-value < 0.05 *vs*. control, **p-value < 0.01 *vs*. control.

Trx proteins provide protection during oxidative stress, due to their antioxidant properties [15]. However, 4-HNE is known to target disulfide bonds, a hallmark of thyroxine containing proteins [13, 16]. To further explain the process by which 4-HNE induces cellular and mitochondrial damage, we measured total Trx in SAECs. SAECs were treated with 25μM 4-HNE and total Trx was measured at 10, 20, 30, and 60 min (Figure 7A). Compared to vehicle-treated controls, our results indicate a significant 40% reduction of total Trx at 30 min, as well as 60% reduction in total Trx at 60 min. Although total Trx was similar to baseline conditions at 10 min, exposure to 4-HNE over time further reduced total Trx. These results suggest that 4-HNE modifies Trx proteins and impairs their function within the cell. We additionally investigated Trx activity in SAECs. Cells were similarly treated with 25 μM 4-HNE and compared to vehicle-treated controls. Total Trx activity was measured at 10, 20, 30, and 60 min time intervals (Figure 7B). There was a time-dependent decrease in Trx activity in response to 4-HNE indicative of protein modification. Trx activity was significantly lowered by 44% at 20 min, with an additional suppression of 63% at 30 min. After 1 h Trx activity decreased by 80% relative to the vehicle-treated controls. Our results provide strong evidence that 4-HNE structurally modifies Trx by targeting cysteine residues, ultimately impacting Trx functionality.

## DISCUSSION

Oxidative stress mediated by the production of ROS and 4-HNE is associated with the pathogenesis of lung disease, such as acute lung injury [29]. Although 4-HNE is endogenously produced and metabolized, under oxidative stress the intrinsic cellular detoxifying responses become overwhelmed and oxidized lipids begin to accumulate, inducing cellular injury [6, 12, 30]. Furthermore, it is known that antioxidants, such as Trx proteins, serve a protective role by responding to oxidants and directly reducing oxidized species [13, 14]. However, the accumulation of reactive lipid by-products impairs the mitochondrial capacity to compensate for the increased energy demand [8]. Consequently, the relation between hyperoxia, 4-HNE and mitochondrial dysfunction warrants further investigation in order to ameliorate mechanisms of lung injury.

We investigated the effects of hyperoxic conditions *in vivo* and found significant increases in 4-HNE mediated protein modification of mice lung tissue after prolonged exposure time of 48 and 72 hours. Previously we have shown that hyperoxic conditions result in alveolar protein leak, pulmonary edema, and increased lung ceramide levels, representative of the damaging responses in acute lung injury [31]. *In vivo* studies have shown that induced hyperoxic conditions by direct mechanisms (i.e. treatment with 4-HNE) or by secondary mechanisms (e.g. cardiac inhibition), stimulate the production of 4-HNE-Protein adducts [10, 11]. Ultimately, protein modification will disrupt the normal functioning of cells, impairing their ability to mediate the response to oxidative stress and ROS production [4]. Inability of proteins to function at desirable capacity will contribute to overall cellular dysfunction and death. Additionally, we have shown an increase in MDA levels in mice lung homogenates after 72 h. The concentration of 4-HNE-protein adducts and MDA have been shown to be highly correlated in samples of human plasma/serum obtained from obese patients, when compared to controls, signifying that these molecules serve as biomarkers for oxidative stress [4].

For the first time, we provide evidence of the damaging effects of 4-HNE in human SAECs, which have been understudied. In response to hyperoxic lung injury and additional pulmonary diseases, such as asthma and chronic obstructive pulmonary disease (COPD), epithelial cells are vulnerable to damage [32]. The function of these cells is essential for preserving the pathway for air to and from the alveoli, and combatting inhaled environmental particulate matter and pathogens [19, 32, 33]; thus, they serve a critical role in protecting against lung injury. Results of our cell viability assays demonstrate 4-HNE-induced SAEC dysfunction. Oxidative stress is known to promote mechanisms of cellular dysfunction, such as DNA fragmentation, ATP depletion, rupture of the cell membrane, as well as apoptosis [7, 9]. Stimulation of apoptosis signaling and pro-apoptotic pathways, such as p53, is known to be induced by various oxidants and 4-HNE [3, 27, 34, 35]. Lipid peroxidation reactions compromise cellular membrane integrity and can mediate apoptosis [36], which is supported by our findings of reduced cellular viability and increased activity of cleaved caspase-3, a marker of apoptosis. Previously, we have shown *in vivo* a suppression of caspase-3 levels in response to the deletion of NLRP3, involved in inflammasome activation, which attenuated hyperoxia-induced oxidative damage [26]. Furthermore, inflammatory responses, including the release of proinflammatory cytokines, are also associated with proapoptotic molecules (i.e. *BAX* or BCL-2 associated protein) and cell death induced by hyperoxic lung injury [31]. In response to the production of reactive aldehydes, epithelial cells can experience an aggregation of leukocytes to the lung airspaces, subsequently promoting an increase in inflammation [8, 37]. 4-HNE has been shown to induce the expression of inflammatory factors, such as COX-2, and regulate the transcription factor NF-kB which further stimulates the expression of inflammatory cytokines [36, 38, 39]. Thus, 4-HNE is associated with various processes of cellular dysfunction and tissue damage, and can contribute to the pathogenesis of disease.

It has been well established that oxidative stress and the generation of reactive aldehydes affect mitochondrial function [11, 21]. Necessary processes of mitochondria, such as oxygen consumption and ATP synthesis become impaired when compensating for the increased energy demand during oxidative stress [40]. Our current findings also support impaired mitochondrial function by reduced ATP levels, Aconitase activity and mitochondrial membrane potential, and inhibited mitochondrial OCR and reserve capacity. Reduction of ATP levels suggest incapacity of SAECs to meet energy demands and decreased respiration efficiency. Furthermore, the ability of SAECs to detoxify 4-HNE is diminished since these processes require energy as well [8]. Damage to mitochondrial membrane protein is devastating for cellular survival since the mitochondrial respiratory chain (complexes I-V) is essential for ATP synthesis, control of electron flow, and production of reducing equivalents [9, 40]. Mitochondrial reserve capacity is considered to be exceptionally important when responding to stress, acting as an additional mechanism in which energy demands can be met, and if exhausted will contribute to protein modification and cell death. This is reinforced by our findings of 4-HNE-Protein adducts and reduced cellular viability [8, 40]. Consequently, 4-HNE inhibits mitochondrial respiration, suggestive of its role in impairing mitochondrial function, and inducing apoptosis and cellular death. Furthermore, addition of ROS inhibitors to SAECs revealed specificity of ROS production to the mitochondrial respiratory chain complex I. Previous studies corroborate that 4-HNE mediated ROS release is associated with mitochondrial respiratory chain complex 1 [41]. Generation of ROS by mitochondrial membrane protein complex I can easily affect mitochondrial function since these oxidized species do not have to be transported from the cytosol [41, 42]. Evidence of the attenuation of ROS production in response to rotenone has been provided previously, which further indicates that respiratory complex 1 is a major source of ROS release [42]. Conversely, other studies suggest that the release of mitochondrial ROS was induced in cells treated with rotenone [43]. However, these studies did not specifically study hyperoxia or 4-HNE induced oxidative stress. This may suggest that further research is required to elucidate the exact mechanisms of rotenone regarding ROS release and 4-HNE, and the possibility that rotenone is mediated by other processes or molecules.

To counteract mitochondrial ROS, we investigated the effects of 4-HNE on Trx proteins in SAECs. Total Trx and Trx activity was found to be reduced in a time-dependent response, suggesting protein modification. In response to hyperoxic lung injury, it has been demonstrated that higher expressions of Trx-dependent mechanisms provide resistance to hyperoxia [13]. Trx proteins, such as mitochondrial Trx2 can metabolize reactive hydrogen peroxide species, providing an antioxidant response [14]. In agreement with other studies, induced oxidative stress by 4-HNE caused cell death, yet Trx2 served a protective role by maintaining mitochondrial function and cell viability, while also inhibiting Hsp70 expression, a protein upregulated in response to oxidative stress [17]. Intracellular loss of Trx levels in our results indicate that ubiquitination and proteasome degradation may be involved in cellular and mitochondrial damage mediated by 4-HNE. This supports previous research on protein degradation by 4-HNE, which show that enzyme functionality becomes inhibited and protein conformation can potentially be altered in the presence of 4-HNE [4]. Our current findings of 4-HNE-Protein adduct formation additionally supports 4-HNE mediated protein degradation. Therefore, inhibition of Trx functionality supports our findings of reduced cellular function, ATP levels, oxygen consumption, and increased mitochondrial ROS production.

In summary, our findings demonstrate for the first time that 4-HNE elicits damaging effects on human SAECs. We show that hyperoxic conditions increased the production of 4-HNE-Protein adducts and MDA levels, signifying their importance as biomarkers of oxidative stress. 4-HNE was shown to reduce SAEC viability and upregulate cleaved caspase-3 activity, a marker of apoptosis. Mitochondrial function was impaired, which reduced oxygen consumption, Aconitase activity, and mitochondrial reserve capacity. JC-1 staining indicated reduced mitochondrial membrane potential in response to 4-HNE. SAEC total ROS production was increased in the presence of 4-HNE and shown to be associated with mitochondrial respiratory chain complex I. Furthermore, 4-HNE inhibited the activity of total Trx proteins, suggesting binding by 4-HNE, and further impairing mitochondrial protective antioxidant properties. Because oxidative stress is involved in the pathogenesis of many diseases and lung epithelial cells are particularly susceptible to oxidative cellular injury [22], targeting 4-HNE in SAECs may provide a novel therapeutic approach to ameliorate hyperoxic lung injury. Taken together, our results signify the importance of remediating 4-HNE induced oxidative cellular and mitochondrial damage within SAECs, in order to maintain intrinsic biological function for cellular survival.

## MATERIALS AND METHODS

### Ethics statement

This study was reviewed and approved by the Institutional Review Board (IRB) of the University of South Florida. Animal studies complied with the Institutional Animal Care and Use Committee (IACUC) protocols and regulations of the University of South Florida.

### Mice studies

C57BL/6J (*N* = 6; wild-type, 6 weeks old, 50% male and 50% female) mice were maintained in a specific-pathogen-free animal facility at the University of South Florida. C57BL/6J mice were purchased from Harlan laboratories (Indianapolis, IN).

### Hyperoxia exposure

C57BL/6J mice (6 weeks old; 20-26 gm) were placed in cages in an airtight chamber (75 × 50 × 50 cm) and exposed to room air (normoxia) or 100% O_2_ for 24, 48, and 72 hours. Oxygen concentration was checked and controlled with proOx P100 sensory (BioSpherix) device, as previously described [31].

### Reagents

Cell culture growth media, fetal bovine serum (FBS), and buffers were purchased from Life Technologies, Inc. (Grand Island, NY) or Lonza (Walkersville, MD). 4-HNE was purchased from (Cayman Chemical Company, Ann Arbor, MI). All other reagents were purchased from Sigma (St Louis, MO). A BCA assay kit was used to determine and quantify the protein concentration of cell lysates (Thermo scientific, Rockford, IL). Microscope slides, ethanol, and all other solvents were from Fisher Scientific (Houston, TX).

### Measurement of 4-HNE-Protein adducts and MDA levels

4-HNE Protein adducts and MDA levels were measured in mice to determine whether toxic lipid peroxidation aldehydes would be produced in response to hyperoxia-induced oxidative stress. C57BL/6J mice (*N* = 6) were exposed to normoxia, as well as hyperoxia for 24, 48, and 72 hours. Lungs were isolated and perfused with cold 0.9% NaCl with 0.1% glucose, 30 mM HEPES, 6mM KCl, 0.1 mg/ml streptomycin sulfate, 0.07 mg/ml penicillin G, 0.07 mg/ml EGTA, and 20 mM Tris-HCl (pH 7.4), as previously described [9]. Homogenates were then centrifuged at 3,000g for 10 min at 4°C and 200 μL of the supernatant was removed. 4-HNE-Protein adduct levels were measured by an OxiSelect HNE-His Adduct ELISA kit, according to manufacturer's instructions (Cell Biolabs, Inc., San Diego, CA). On a 96-well plate, protein samples and standards (10 μg/ml) were absorbed for 2 hrs at 37°C. Protein was probed with anti-HNE Histidine (His) antibody, followed by horseradish peroxidase secondary antibody. Protein levels were compared with a standard curve produced from HNE-BSA standards. MDA levels were measured in mice lung homogenates with a Lipid Peroxidation (MDA) Assay Kit (Colorimetric/Fluorometric) ab118970 (abcam, Cambridge, MA), after washing with cold PBS. MDA levels were also compared with a MDA standard curve, prepared from MDA Standard.

### Cell culture

SAECs were maintained in SABM medium (Lonza, Walkersville, MD) supplemented with 10% FBS, Penicillin (100 u/ml) Streptomycin (100 μg/ml) and Clonetics SAGM SingleQuots (Lonza) at 37°C in a 5% CO_2_ humidified incubator, in order to maintain adequate cell growth. Cultured cells deemed confluent were treated with 5, 10, and/or 25 μM of 4-HNE, respective to the experiment, every 3 hours. Cells were incubated for an additional hour to perform cell viability assays and JC-1 staining.

### Cell viability assays

MTT [3,-(4,5-dimethylthiazol-2-yl)-2,5-diphenyltetrazolium bromide] assay was used to assess cell viability, according to methods previously described [37, 44]. Briefly, SAECs were treated with 5, 10, and 25 μM 4-HNE, then incubated for 1 hour. Cells were then treated with yellow MTT (0.5 mg/ml) per well and incubated for 2 hours at 37°C. Medium was removed, and resultant purple formazan crystals were dissolved in 250μl DMSO. Absorbance of formazan products was measured at 570nm. Trypan Blue Exclusion Assay protocol was described previously [45]. SAECs were treated with 5, 10, and 25 μM 4-HNE, then incubated for 1 hour. Cells were then treated with Trypan blue dye (0.04% in PBS) after exposure to H_2_O_2_ for 1 hour, placed on a hemocytometer to determine cell count, and examined by light microscopy. Viable and dead (blue) cells were counted out of a total of 200 cells, with results expressed as percentage of viable cells.

### Determination of caspase-3 activity

Cleaved Caspase-3 activity was measured in SAECs treated with 5-25 μM 4-HNE and assessed using a colorimetric assay kit (R&D systems, Minneapolis, MN, USA), as per manufacturer's guidelines. Caspase-3 colorimetric substrate, DEV-pNA, was used to perform the enzymatic reaction in 96 well plates at 37°C, as previously described [46]. Caspase activity was determined by using a micro-plate reader.

### Quantification of percent ATP levels

SAECs were treated with 5, 10, and 25 μM 4-HNE and compared to control cells that were treated with vehicle (0.1% ethanol). Percent ATP levels were measured using a bioluminescent assay (Berthold Systems, Aliquippa, PA) with recombinant firefly luciferase and its substrate D-luciferin (Molecular Probes, Eugene, OR), as described previously [25]. Light produced was detected by a luminometer (Berthold Systems, Aliquippa, PA).

### Aconitase assay

Mitochondrial Aconitase activity was determined in cell lysates using an Aconitase Assay kit (OxisResearch, Foster City, CA), according to manufacturer's instructions and methods previously described elsewhere [27, 28]. Aconitase activity was represented in μU/min/mg of mitochondrial protein. Conversion of citrate into isocitrate was determined by measuring absorbance at 340 nm for 30 min at 37°C, and monitoring NADPH production.

### Oxygen consumption rate

Bioenergetic parameters were evaluated by using XF analysis (Seahorse Biosciences, Chicopee, MA), as previously described [8, 40]. Briefly, one hour before assay measurements were taken, culture medium was removed and replaced with unbuffered Dulbecco's modified Eagles/medium (pH 7.4). After baseline oxygen consumption rate (OCR) measurements (pmol/O_2_/min), oligomycin (1 μg/ml), FCCP (1μM), and antimycin A (10 μM) were injected consecutively through Seahorse Flux Pak cartridges and documented after each injection. Oligomycin was used to measure non-ATP linked OCR, FCCP was used to determine maximal mitochondrial respiration, and antimycin A was used to measure non-mitochondrial OCR.

### Mitochondrial JC-1 staining

SAECs were maintained in SABM medium (Lonza) supplemented with 10% FBS, Penicillin (100 u/ml) Streptomycin (100 μg/ml) and Clonetics SAGM SingleQuots (Lonza) at 37°C in a 5% CO_2_ humidified incubator. The cells were plated at a density of 2 × 10^5^ cells/ml in a 12 mm Nunc glass bottom dish (Thermo Scientific). The cells were treated with different concentration of 4-hydroxynonenal (4-HNE) (10-100 μM) or vehicle (0.1% ethanol) at 37°C for 15 min. Following washes, cells were incubated with 10 μg/ml of JC-1 (Molecular Probes, Invitrogen) at 37°C for 15 min and cells were washed three times and placed in the constituted medium. We performed live cell imaging using 3i Spinning disk confocal microscope (Olympus, Pittsburgh, PA). The monomer was excited by 488 nm argon-ion laser, whereas the J-aggregate forms were excited selectively using the 568 argon-krypton laser line. Polarized mitochondria were visualized as punctate orange red fluorescence. On depolarization, the fluorescence changed from orange red to green. The images were processed using Adobe Photoshop CS6. Magnification = 60X. Image J software was used to quantify JC-1 aggregates, with mean intensity expressed as Arbitrary Units.

### Measurement of Total ROS

ROS production (hydroxyl, peroxyl, other ROS) was evaluated in SAECs by using an OxiSelect Intracellular ROS Assay Kit (Cell Biolabs, Inc., San Diego, CA). Cells were cultured in a 96-well plate and oxidation of the cell permeable fluorescent probe 2′, 7′-Dichlorodihydrofluorescin diacetate (DCFH-DA) to 2′, 7′-Dichlorodihydrofluorescein (DCF) by ROS was measured. Cells were treated with 10 μM DCFH-DA for 30 min in the absence of light at room temperature, as previously described [27]. Fluorescence intensity was proportional to the ROS levels within the cell cytosol and compared to a DCF standard curve. A fluorescence microplate reader was used to measure absorbance at 480 nm excitation and 530 nm emission. Inhibitors, 1mM apocynin, 100 μM allopurinol and 1 μM rotenone, were added to study their effects within SEACs and the mitochondria.

### Trx quantification

Trx levels were quantified using an Enzyme Linked Immunoassay (ELISA) kit (Cedarlane Laboratories, Burlington, NC), according to manufacturer's instructions. Briefly, lung protein extracts was immobilized on a 96-well plate, then blocked for 1 hour at room temperature with blocking buffer and 0.05% Tween 20. Plates were washed with Tris-buffered saline and phosphate buffered saline, containing 0.05% Tween 20. Antigen was probed with primary monoclonal anti-HNE antibody (Alpha Diagnostic Intl, San Antonio, TX) diluted 1000 fold.

### Determination of Trx activity

Trx activity was determined enzymatically in cultured cells, according to methods described elsewhere [47, 48], including TrxR activity for comparison. 100 μl of 10.1% Triton X-100/PBS was used to induce cell lysis and a 5 μl aliquot was analyzed for protein concentration using BioRad protein assay (Hercules, CA), with bovine serum albumin (BSA) included as the standard. Protein was combined with 137 μl of reaction mixture [0.1 M Tris-HCl (pH 7.5), 2 mM EDTA, 0.2 mM NADPH, and 75 nM rat TR]. Insulin-reducing assay was used, according to methods previously described, to determine Trx activity [48]. The reaction was evaluated by spectrophotometric analysis, recording the decrease in absorbance at 340 nm each 30 seconds in a 5 minute time span, to monitor the oxidation of NADPH. The control samples were subjected to the same treatment, except in the absence of insulin. ΔA_340_ of control samples were subtracted from the ΔA_340_ of each experimental sample. Calculated values were plotted on a standard curve to determine the absolute amounts of Trx in the cell extract.

### Statistical analysis

In all *in vivo* experiments, we included *N* = 6 mice per group. Values are expressed as means ± SE. Statistical significance was calculated by using Windows GraphPad Prism version 10.00 (GraphPad Software, San Diego, CA). Between group differences were determined using Student's unpaired *t*-test. Comparison of variables between three or more groups was performed using one-way ANOVA followed by post-hoc Tukey HSD test. All tests were two-tailed and p-values less than 0.05, 0.01, and 0.001 were considered statistically significant.

## References

[1] Kallet RH, Matthay MA (2013). Hyperoxic acute lung injury. Respiratory care.

[2] Bhakta KY, Jiang W, Couroucli XI, Fazili IS, Muthiah K, Moorthy B (2008). Regulation of cytochrome P4501A1 expression by hyperoxia in human lung cell lines: Implications for hyperoxic lung injury. Toxicology and applied pharmacology.

[3] Crapo JD (1986). Morphologic changes in pulmonary oxygen toxicity. Annual review of physiology.

[4] Weber D, Milkovic L, Bennett SJ, Griffiths HR, Zarkovic N, Grune T (2013). Measurement of HNE-protein adducts in human plasma and serum by ELISA-Comparison of two primary antibodies. Redox biology.

[5] Chaudhary P, Sharma R, Sahu M, Vishwanatha JK, Awasthi S, Awasthi YC (2013). 4-Hydroxynonenal induces G2/M phase cell cycle arrest by activation of the ataxia telangiectasia mutated and Rad3-related protein (ATR)/checkpoint kinase 1 (Chk1) signaling pathway. The Journal of biological chemistry.

[6] Negre-Salvayre A, Coatrieux C, Ingueneau C, Salvayre R (2008). Advanced lipid peroxidation end products in oxidative damage to proteins. Potential role in diseases and therapeutic prospects for the inhibitors. British journal of pharmacology.

[7] Pagano A, Barazzone-Argiroffo C (2003). Alveolar cell death in hyperoxia-induced lung injury. Annals of the New York Academy of Sciences.

[8] Hill BG, Dranka BP, Zou L, Chatham JC, Darley-Usmar VM (2009). Importance of the bioenergetic reserve capacity in response to cardiomyocyte stress induced by 4-hydroxynonenal. The Biochemical journal.

[9] Waxman AB, Kolliputi N (2009). IL-6 protects against hyperoxia-induced mitochondrial damage *via* Bcl-2-induced Bak interactions with mitofusins. American journal of respiratory cell and molecular biology.

[10] Hill BG, Haberzettl P, Ahmed Y, Srivastava S, Bhatnagar A (2008). Unsaturated lipid peroxidation-derived aldehydes activate autophagy in vascular smooth-muscle cells. The Biochemical journal.

[11] Gomes KM, Campos JC, Bechara LR, Queliconi B, Lima VM, Disatnik MH, Magno P, Chen CH, Brum PC, Kowaltowski AJ, Mochly-Rosen D, Ferreira JC (2014). Aldehyde dehydrogenase 2 activation in heart failure restores mitochondrial function and improves ventricular function and remodelling. Cardiovascular research.

[12] Chen L, Zong R, Zhou J, Ge L, Zhou T, Ma JX, Liu Z, Zhou Y (2015). The oxidant role of 4-hydroxynonenal in corneal epithelium. Scientific reports.

[13] Tipple TE, Welty SE, Rogers LK, Hansen TN, Choi YE, Kehrer JP, Smith CV (2007). Thioredoxin-related mechanisms in hyperoxic lung injury in mice. American journal of respiratory cell and molecular biology.

[14] Kudin AP, Augustynek B, Lehmann AK, Kovacs R, Kunz WS (2012). The contribution of thioredoxin-2 reductase and glutathione peroxidase to H(2)O(2) detoxification of rat brain mitochondria. Biochimica et biophysica acta.

[15] Lee S, Kim SM, Lee RT (2013). Thioredoxin and thioredoxin target proteins: from molecular mechanisms to functional significance. Antioxidants & redox signaling.

[16] Fang J, Holmgren A (2006). Inhibition of thioredoxin and thioredoxin reductase by 4-hydroxy-2-nonenal *in vitro* and *in vivo*. Journal of the American Chemical Society.

[17] Sugano E, Murayama N, Takahashi M, Tabata K, Tamai M, Tomita H (2013). Essential role of thioredoxin 2 in mitigating oxidative stress in retinal epithelial cells. Journal of ophthalmology.

[18] Carbone DL, Doorn JA, Petersen DR (2004). 4-Hydroxynonenal regulates 26S proteasomal degradation of alcohol dehydrogenase. Free radical biology & medicine.

[19] Tesfaigzi Y (2006). Roles of apoptosis in airway epithelia. American journal of respiratory cell and molecular biology.

[20] Barker GF, Manzo ND, Cotich KL, Shone RK, Waxman AB (2006). DNA damage induced by hyperoxia: quantitation and correlation with lung injury. American journal of respiratory cell and molecular biology.

[21] Kolliputi N, Waxman AB (2009). IL-6 cytoprotection in hyperoxic acute lung injury occurs *via* suppressor of cytokine signaling-1-induced apoptosis signal-regulating kinase-1 degradation. American journal of respiratory cell and molecular biology.

[22] Mach WJ, Thimmesch AR, Pierce JT, Pierce JD (2011). Consequences of hyperoxia and the toxicity of oxygen in the lung. Nursing research and practice.

[23] Vyas-Read S, Wang W, Kato S, Colvocoresses-Dodds J, Fifadara NH, Gauthier TW, Helms MN, Carlton DP, Brown LA (2014). Hyperoxia induces alveolar epithelial-to-mesenchymal cell transition. American journal of physiology Lung cellular and molecular physiology.

[24] Elmer J, Scutella M, Pullalarevu R, Wang B, Vaghasia N, Trzeciak S, Rosario-Rivera BL, Guyette FX, Rittenberger JC, Dezfulian C (2015). The association between hyperoxia and patient outcomes after cardiac arrest: analysis of a high-resolution database. Intensive care medicine.

[25] Kolliputi N, Shaik RS, Waxman AB (2010). The inflammasome mediates hyperoxia-induced alveolar cell permeability. Journal of immunology (Baltimore, Md : 1950).

[26] Fukumoto J, Fukumoto I, Parthasarathy PT, Cox R, Huynh B, Ramanathan GK, Venugopal RB, Allen-Gipson DS, Lockey RF, Kolliputi N (2013). NLRP3 deletion protects from hyperoxia-induced acute lung injury. American journal of physiology Cell physiology.

[27] Perrino C, Feliciello A, Schiattarella GG, Esposito G, Guerriero R, Zaccaro L, Del Gatto A, Saviano M, Garbi C, Carangi R, Di Lorenzo E, Donato G, Indolfi C, Avvedimento VE, Chiariello M (2010). AKAP121 downregulation impairs protective cAMP signals, promotes mitochondrial dysfunction, and increases oxidative stress. Cardiovascular research.

[28] Yan LJ, Levine RL, Sohal RS (1997). Oxidative damage during aging targets mitochondrial aconitase. Proceedings of the National Academy of Sciences of the United States of America.

[29] Chow CW, Herrera Abreu MT, Suzuki T, Downey GP (2003). Oxidative stress and acute lung injury. American journal of respiratory cell and molecular biology.

[30] Usatyuk PV, Natarajan V (2004). Role of mitogen-activated protein kinases in 4-hydroxy-2-nonenal-induced actin remodeling and barrier function in endothelial cells. The Journal of biological chemistry.

[31] Kolliputi N, Galam L, Parthasarathy PT, Tipparaju SM, Lockey RF (2012). NALP-3 inflammasome silencing attenuates ceramide-induced transepithelial permeability. Journal of cellular physiology.

[32] Crystal RG, Randell SH, Engelhardt JF, Voynow J, Sunday ME (2008). Airway epithelial cells: current concepts and challenges. Proceedings of the American Thoracic Society.

[33] Pirela SV, Miousse IR, Lu X, Castranova V, Thomas T, Qian Y, Bello D, Kobzik L, Koturbash I, Demokritou P (2015). Effects of Laser Printer-Emitted Engineered Nanoparticles on Cytotoxicity, Chemokine Expression, Reactive Oxygen Species, DNA Methylation, and DNA Damage: A Comprehensive Analysis in Human Small Airway Epithelial Cells, Macrophages, and Lymphoblasts. Environmental health perspectives.

[34] Li J, Sharma R, Patrick B, Sharma A, Jeyabal PV, Reddy PM, Saini MK, Dwivedi S, Dhanani S, Ansari NH, Zimniak P, Awasthi S, Awasthi YC (2006). Regulation of CD95 (Fas) expression and Fas-mediated apoptotic signaling in HLE B-3 cells by 4-hydroxynonenal. Biochemistry.

[35] Sharma A, Sharma R, Chaudhary P, Vatsyayan R, Pearce V, Jeyabal PV, Zimniak P, Awasthi S, Awasthi YC (2008). 4-Hydroxynonenal induces p53-mediated apoptosis in retinal pigment epithelial cells. Archives of biochemistry and biophysics.

[36] Yadav UC, Ramana KV (2013). Regulation of NF-kappaB-induced inflammatory signaling by lipid peroxidation-derived aldehydes. Oxidative medicine and cellular longevity.

[37] Jinsmaa Y, Florang VR, Rees JN, Anderson DG, Strack S, Doorn JA (2009). Products of oxidative stress inhibit aldehyde oxidation and reduction pathways in dopamine catabolism yielding elevated levels of a reactive intermediate. Chemical research in toxicology.

[38] Park S, Sung B, Jang EJ, Kim DH, Park CH, Choi YJ, Ha YM, Kim MK, Kim ND, Yu BP, Chung HY (2013). Inhibitory action of salicylideneamino-2-thiophenol on NF-kappaB signaling cascade and cyclooxygenase-2 in HNE-treated endothelial cells. Archives of pharmacal research.

[39] Zarrouki B, Soares AF, Guichardant M, Lagarde M, Geloen A (2007). The lipid peroxidation end-product 4-HNE induces COX-2 expression through p38MAPK activation in 3T3-L1 adipose cell. FEBS letters.

[40] Sansbury BE, Jones SP, Riggs DW, Darley-Usmar VM, Hill BG (2011). Bioenergetic function in cardiovascular cells: the importance of the reserve capacity and its biological regulation. Chemico-biological interactions.

[41] Usatyuk PV, Parinandi NL, Natarajan V (2006). Redox regulation of 4-hydroxy-2-nonenal-mediated endothelial barrier dysfunction by focal adhesion, adherens, and tight junction proteins. The Journal of biological chemistry.

[42] Pignatelli M, Sanchez-Rodriguez J, Santos A, Perez-Castillo A (2005). 15-deoxy-Delta-12,14-prostaglandin J2 induces programmed cell death of breast cancer cells by a pleiotropic mechanism. Carcinogenesis.

[43] Li N, Ragheb K, Lawler G, Sturgis J, Rajwa B, Melendez JA, Robinson JP (2003). Mitochondrial complex I inhibitor rotenone induces apoptosis through enhancing mitochondrial reactive oxygen species production. The Journal of biological chemistry.

[44] Mosmann T (1983). Rapid colorimetric assay for cellular growth and survival: application to proliferation and cytotoxicity assays. Journal of immunological methods.

[45] Kolliputi N, Waxman AB (2009). IL-6 cytoprotection in hyperoxic acute lung injury occurs *via* PI3K/Akt-mediated Bax phosphorylation. American journal of physiology Lung cellular and molecular physiology.

[46] Raza H, John A (2012). Implications of altered glutathione metabolism in aspirin-induced oxidative stress and mitochondrial dysfunction in HepG2 cells. PloS one.

[47] Sasada T, Nakamura H, Ueda S, Sato N, Kitaoka Y, Gon Y, Takabayashi A, Spyrou G, Holmgren A, Yodoi J (1999). Possible involvement of thioredoxin reductase as well as thioredoxin in cellular sensitivity to cis-diamminedichloroplatinum (II). Free radical biology & medicine.

[48] Yang X, Wu X, Choi YE, Kern JC, Kehrer JP (2004). Effect of acrolein and glutathione depleting agents on thioredoxin. Toxicology.

